# Editorial: STATs and IRFs in Innate Immunity: From Transcriptional Regulators to Therapeutic Targets

**DOI:** 10.3389/fimmu.2019.01829

**Published:** 2019-08-06

**Authors:** Chien-Kuo Lee, Hans A. R. Bluyssen

**Affiliations:** ^1^Graduate Institute of Immunology, National Taiwan University College of Medicine, Taipei, Taiwan; ^2^Department of Human Molecular Genetics, Faculty of Biology, Institute of Molecular Biology and Biotechnology, Adam Mickiewicz University, Poznań, Poland

**Keywords:** STAT, IRF, innate immunity, transcription regulation, therapeutic target

Signal Transducer and Activator of Transcription (STAT) and Interferon Regulatory Factor (IRF) are important transcriptional regulators that modulate crucial aspect of innate and adaptive immunity. Among their activating signals are cytokines and growth factors, including interferons (IFNs), interleukins (ILs), and growth factors like EGF and PDGF. Also many oncogenic signals and pathogenic responses, dependent on pattern-recognition receptors (PRRs), are among STAT and IRF activators.

STATs facilitate action of cytokines, growth factors, and pathogens, mainly through membrane receptor-associated Janus kinases (JAK). The STAT family is composed of seven members, namely STAT1, STAT2, STAT3, STAT4, STAT5A, STAT5B, and STAT6. Structurally they share five domains, which are an amino-terminal domain, a coiled-coil domain, a DNA-binding domain, an SH2 domain and a carboxy-terminal transactivation domain. STAT activation is mediated by a highly conserved SH2 domain, which interacts with phosphotyrosine (pTyr) motifs for specific STAT-receptor contacts and STAT dimerization. The active dimers induce gene transcription in the nucleus by binding to a specific DNA-response element (TTCN_2−4_ GAA) of target genes.

IRFs are primarily related to the innate response of the immune system that is dependent on PRRs, including Toll-Like Receptor (TLR)s. IRFs comprise a family of nine homologous proteins (IRF1–9), which contain a conserved DNA binding domain and IRF association domain. The DNA binding domain is located at the amino termini of IRFs and consists of a five-tryptophan repeat that recognizes a DNA motif-IFN regulatory element (IRE, NAANNGAAA) or its tandem-repeat form called the IFN-stimulated response element (ISRE, A/GNGAAANNGAAACT), present in the regulatory regions of IFNs and IFN-inducible genes (ISGs). The C-terminal halve contains an IRF association domain (IAD), with which they interact with IRF family members, other transcription factors, or self-associate, which is crucial during DNA binding. These interactions allow IRFs to modulate their activity and bind a variety of target genes.

Genome-wide transcription profiling and chromatin association studies identified many STAT and IRF targets, including protein-coding and non-coding genes like microRNAs and long non-coding RNAs. In addition, complex transcriptional regulatory mechanisms have been identified that predict co-binding strategies of STATs and IRFs that are at the basis of important immuno-regulatory and oncogenic responses.

Finally, abnormalities in activation of STAT- and IRF-dependent pathways as well as genetic mutations appear in many diseases like: viral infections, autoimmune diseases, cardiovascular diseases, asthma and allergies, and cancer, consequently identifying these proteins as highly interesting therapeutic targets.

This special issue is a collection of 1 mini-review, 8 reviews, 1 hypothesis and theory article, and 5 research articles. The first article of this Research Topic by Mogensen provides a detailed overview of STAT- and IRF-dependent signaling pathways activated by type I and type II IFNs, but also other cytokines and growth factors and PRRs. In addition, an overview is presented of their essential role in PRR-mediated type I IFN production, as a hallmark of human immune defenses toward microbial pathogens, particularly viruses. Moreover, Mogensen summarizes the infectious, inflammatory, and autoimmune disorders arising from human inborn errors caused by gain- and loss-of-function mutations in IRFs and STATs. Loss-of-function mutations of IRFs, including IRF3, IRF7, IRF8 and IRF9 and STATs including STAT1, STAT2 and STAT3, result in primary immunodeficiencies with increased susceptibility to infections with viruses, bacteria and fungi, while gain-of-function mutations of some of these factors lead to autoimmunity and auto-inflammation, demonstrating the underlying mechanisms of pathogenesis and providing therapeutic potentials targeting these molecules.

With their primary roles in immunity, STATs and IRFs are also known to participate in regulatory networks controlling inflammation. Cells engaging in inflammation undergo drastic changes of their transcriptomes. In order to tailor these alterations in gene expression to the requirements of the inflammatory process, tight and coordinate regulation of gene expression by environmental cues, microbial or danger-associated molecules or cytokines, are mandatory. Platanitis and Decker describe the complex role of STATs, IRFs, and nuclear factor kB (NFkB), in collaboration with pioneer or lineage determining transcription factors (LDTFs) as critical determinants of the changes in chromatin landscapes and transcriptomes that specify potential consequences of inflammation: tissue repair, training, and tolerance.

STAT1 is a shared signal mediator of type I and type II IFN to regulate innate and adaptive immunity. The functions of STAT1, like other STAT proteins, are dictated by its C-terminal transactivation domain (TAD) through recruiting transcriptional co-activators. However, the detailed mechanisms remain to be elucidated. Parrini et al. used primary macrophages expressing only STAT1β that lacks TAD to demonstrate that TAD of STAT1 is required for recruitment of the core components of Mediator complex on the promoter of IRF1 and IRF8, which harbors open chromatin state at basal conditions. Intriguingly, STAT1 TAD is dispensable for IFNγ-mediated expression of IRF7, which is mediated by STAT1 in complex with STAT2 and IRF9, suggesting that there is a novel function of TAD and a gene-specific transcription activity of STAT1β.

Gene expression regulation of many pro-inflammatory genes has shown to rely on Signal Integration (SI) between IFNs and TLR4 through combinatorial actions of STAT1 and NFκB. Thus, IFN pre-treatment (“priming”) followed by LPS stimulation leads to enhanced transcriptional responses as compared to the individual stimuli. Piaszyk-Borychowska et al. characterized the genome-wide mechanism of priming-induced IFNα + LPS– and IFNγ + LPS-dependent SI in vascular smooth muscle cells (VSMCs) as compared to macrophages (MQs) and Dendritic cells (DCs). Thus, they identified IFNα + LPS or IFNγ + LPS induced genes commonly expressed in these cell types that bound STAT1 and p65 at comparable GAS, ISRE, or NFκB sites in promoter proximal and distal regions. Moreover, SI was dependent on epigenetically directed STAT1-p65 co-binding to GAS-NFκB or ISRE-NFκB composite sites, resulting in robust transcriptional activation of pro-inflammatory and pro-atherogenic genes. Piaszyk-Borychowska et al. also offer an explanation for the comparable effects of IFNα or IFNγ priming on TLR4-induced STAT1 activation in vascular and immune cells, with important implications in atherosclerosis.

Based on this, STAT1 represents an interesting therapeutic target for cardiovascular diseases (CVDs), including atherosclerosis. However, due to high sequence homology of the SH2 domain in different STATs, it is difficult to generate STAT1-specific inhibitors. Thus, development of multi-STAT inhibitors is more feasible although it may seem counterintuitive. Nevertheless, it can be a viable strategy to treat inflammatory diseases including atherosclerosis. Plens-Galaska et al. takes advantage of *in silico* docking of the SH2 domain of multi-STAT proteins and identified a novel inhibitor C01L_F03, which simultaneously blocks IFN-I-dependent transcriptional activity of STAT1, STAT2, and STAT3. Moreover, C01L_F03 and two other multi-STAT inhibitors STATTIC and STX-0119 also suppress combined treatment of IFNγ and LPS induced pro-inflammatory and pro-atherogenic gene expression and the functions of human microvascular endothelial cells (HMEC), leukocytes, and mesenteric artery required for atherogenesis. Therefore, these results provide a therapeutic potential of multi-STAT inhibitors in atherosclerosis.

Sepsis is a form of systemic inflammation during bacterial infection, which causes severe morbidity and mortality. It is known that IFN-I production is accompanied by the onset of sepsis. However, the role of IFN-I in the pathogenesis of sepsis remains controversial. McKenna et al. found that LPS-induced endotoxemia induces hepatic p65/NFκB and IRF3 activation, leading to increased production of IFNβ in the serum, signaling of pulmonary STAT1 and expression of its downstream genes in adult mice. The endotoxemia in neonatal mice, on the contrary, reduces p65/NFκB but increases immunotolerant p50/NFκB signaling and impairs IRF3 activation and IFNβ expression. Moreover, IFNβ pre-treatment of endotoxemic neonates results in significant improved survival following challenge with lethal endotoxemia. These results suggest that LPS-induced IFN expression is attenuated in neonates and that there is an age-dependent response in mouse model of sepsis.

While STAT3 is activated by both pro-inflammatory cytokines such as IL-6 and anti-inflammatory cytokines such as IL-10, the biological role of STAT3 is known to be context- and tissue-specific. Kurdi et al. reviewed recent studies on cardiac STAT3 and address the important role of STAT3 in maintaining normal structure and contractile activity during remodeling of cardiomyocytes and is protective in cardiac diseases, including hypertrophy, heart failure, myocardial infarction, peripartum cardiomyopathy, and viral myocarditis. In addition to IL-6 family of cytokines, STAT3 is also activated by IFN-I and appears to suppress IFN-I responses. Tsai et al. reviewed recent progress in the regulatory activity of STAT3 and proposed several mechanisms, including attenuating IFN-I signaling, co-operating with co-repressors, downregulating ISGF3 components and inducing negative regulators. Interestingly, this feedback regulation of STAT protein is evolutionarily conserved in both vertebrates and invertebrates. The negative effect of STAT3 is exploited by several viruses to evade host innate immunity, providing a biological significance of this activity and a therapeutic potential by targeting STAT3 to boost antiviral response and to treat IFN-I-associated diseases.

Subsequently, the review articles of Jefferies and Paul et al. focus on the IRF family of transcription factors of which IRF3, IRF5, and IRF7, are critical to production of type I interferons downstream of pathogen recognition receptors that detect viral RNA and DNA. A fourth family member, IRF9, regulates interferon-driven gene expression as part of the interferon-stimulated gene factor 3 (ISGF3). In addition, IRF4, IRF8, and IRF5 regulate myeloid cell development and phenotype, thus playing important roles in regulating inflammatory responses. First, Jefferies highlights the role of IRF family members in regulating type I IFN production and responses and myeloid cell development or differentiation, with particular emphasis on how regulation of their levels and activity by ubiquitination and microRNAs may impact autoimmune disease. In addition, Paul et al. more specifically outline the structural basis of IRF9 that guides its regulation and interaction in antiviral immunity and other diseases.

Two other articles, by Thompson et al. and Antonczyk et al. deal with the issue of therapeutic targeting of IRFs in connection to IRF-dependent disorders and malignancies. These articles focus on IRF-dependent transcriptional regulatory mechanisms, accompanied by post-translational modifications, downstream of IFNs, and pattern recognition receptors (PRRs). Identification of disease-specific IRF-target genes could serve as diagnostic markers. Moreover, identification of structural features of the IRFs identify these proteins as interesting therapeutic targets and warrants the development of novel therapeutic strategies. Thus, Thompson et al. describe potential therapeutic strategies for targeting all IRFs by using IRF5 as a candidate targeting molecule. Antonczyk et al. on the other hand, proposes a novel direct IRF-modulating strategy employing a pipeline approach that combines comparative *in silico* docking to the IRF-DNA Binding Domain with *in vitro* validation of IRF inhibition. They hypothesize that this methodology will enable the efficient identification of IRF-specific and pan-IRF inhibitors that can be used for the treatment of IRF-dependent disorders and malignancies.

An example of the diagnostic potential of IRFs is assessed by the research article of Rodriguez-Carrio et al., in which the differential expression of IRF4 and IRGs observed in SLE and RA can delineate gene expression signatures associated with clinical features and treatment outcomes. This study supports a clinically-relevant phenomenon of shaping of the IFN signature by IRF4 in autoimmune patients.

The prominent functions of IFN in antiviral response is mediated by ISGs that are regulated by STATs and IRFs through post-translational modification and complex assembly. However, viruses also evolved many strategies to escape or evade antiviral activity of IFNs by targeting the members of these two families. Chiang and Liu reviewed recent evidence in IRF-mediated IFN production during virus infection and summarize several mechanisms of viral regulation and evasion of IRF- and STAT-dependent antiviral pathways, including disrupting post-translational modifications, inducing proteolytic degradation or relocalization, inhibiting transcriptional complex formation and blocking the expression of IRFs/STATs. Among different viruses, dengue virus (DENV), a single-stranded, positive-sense RNA virus, has long been considered to be a weak IFN-inducing pathogen. It, however, becomes clear that DENV has evolved multiple strategies to subvert innate immunity. Kao et al. reviewed the current knowledge of how DENV escapes innate immunity and outline the tactics of DENV, including targeting both RNA-dependent RLR-MAVS and DNA-dependent cGAS-STING pathways to block IFN-I production and inhibiting IRF and STAT signaling to impede IFN-I action. Gaining insight into mechanisms of the interplays between host and viruses may help develop therapeutic approaches to control viral spread and to avoid life-threatening diseases resulting from viral infections.

In conclusion, this Research Topic provides a comparative overview of STATs and IRFs in innate immunity, with the emphasis on their function as transcriptional regulators during immune-regulatory and oncogenic responses, their pathogenic role in different diseases and their potential as therapeutic targets. We tried to cover the most recent advances in diseases resulting from mutations of STATs and IRFs in mice and humans and in the regulation of type I IFN production and evasion of innate immunity by viruses through these two family members. Understanding the basis of these events may provide strategies for developing therapeutics for the diseases and antiviral responses.

## Author Contributions

HB and C-KL were both involved in writing and editing the manuscript and approved it for publication.

### Conflict of Interest Statement

The authors declare that the research was conducted in the absence of any commercial or financial relationships that could be construed as a potential conflict of interest.

